# Efficacy of innovative polyglycolic acid sheet device delivery station system: a randomized prospective study

**DOI:** 10.1007/s00464-017-6019-6

**Published:** 2018-01-08

**Authors:** Hirohito Mori, Yu Guan, Hideki Kobara, Seiki Kobayashi, Noriko Nishiyama, Nobuya Kobayashi, Tsutomu Masaki

**Affiliations:** 1 Department of Gastroenterology and Neurology, Faculty of Medicine, Kagawa University, 1750-1 Ikenobe, Miki, Kita, Kagawa 761-0793 Japan; 2 Department of Pharmacology, Kagawa University, 1750-1 Ikenobe, Miki, Kita, Kagawa 761-0793 Japan; 3 Department of Gastroenterological Surgery, Ehime Rosai Hospital, 13-27, Minamikomatsubara, Niihama, Ehime 792-8550 Japan

**Keywords:** Delivery of polyglycolic acid sheet, Coating area per minute, Post-endoscopic submucosal dissection bleeding, Device delivery station system

## Abstract

**Background:**

Although there have been several reports of treating large post-endoscopic submucosal dissection (ESD) ulcers by covering them with a polyglycolic acid sheet (PGAs), this approach presents problems regarding PGAs delivery. This study assessed the usefulness of a device delivery station system (DDSS) to evaluate the appropriate and rapid PGAs coating method with DDSS.

**Methods:**

Thirty-nine of 41 patients who were diagnosed with early gastric cancer over 20 mm in diameter and pathologically diagnosed with well-differentiated adenocarcinoma were randomly allocated to the following two groups according to delivery method: the conventional PGAs delivery group (C group) (*n* = 19) and the new DDSS group (DDSS group) (*n* = 20). The primary outcome was the coating area per minute in the C group and DDSS group (cm^2^/min).

**Results:**

There were significant differences in the coating time (min), with values of 34.1 (15.0–60.7) vs. 16.85 (11.5–27.2) min for the C group and DDSS group, respectively (*p* = 0.001). There was also a significant difference in coating area per minute, with values of 0.261 (0.02–1.00) and 0.96 (0.173–2.06) cm^2^/min for the C group and DDSS group, respectively (*p* = 0.001). There were four cases of post-ESD bleeding (1–7 days after ESD) in the C group compared with 0 in the DDSS group, which represented a significant difference (*p* = 0.030).

**Conclusions:**

The DDSS was very useful for rapidly delivering and tightly attaching a PGAs to control post-ESD bleeding.

**Trial registration:**

University Hospital Medical Network (UMIN) 000026377.

**Electronic supplementary material:**

The online version of this article (10.1007/s00464-017-6019-6) contains supplementary material, which is available to authorized users.

There have been several reports of treatments for large, artificial post-endoscopic submucosal dissection (ESD) ulcers, such as natural ulcer healing, ulcer closure using an over-the-scope clip (OTSC) system (Ovesco Endoscopy GmbH, Tüebingen, Germany) [1] and covering an ulcer with a polyglycolic acid sheet (PGAs) (Gunze Co., Kyoto, Japan); treatment with a PGAs has been used in particular to prevent post-ESD bleeding [2, 3]. Although mechanical means of closing larger and deeper mucosal defects via a hemoclip or OTSC system, which reach deeper, submucosal layers after ESD, effectively prevent post-ESD bleeding or delayed perforation to some extent, such mechanical closures create a mucosal bridge over the remaining muscular layer when applied without a coating or covering for larger, deeper defects reaching submucosal layers. Filling and covering a large, deep defect with a PGAs is a more natural and effective way for treating large post-ESD artificial ulcers to prevent post-ESD adverse events, such as bleeding and perforation. It is especially difficult to close artificial ulcers ≥ 5 cm using a hemoclip or OTSC closure. As such, filling and covering large artificial defects with a PGAs is a more ideal treatment approach; however, it is difficult to deliver and thoroughly cover an ulcer floor with a thin PGAs using only a pan-endoscope, especially under wet conditions. PGAs delivery is one of the main problems of treating artificial ulcers. The conventional PGAs coating method is very time consuming and causes maneuverability difficulties [4–8]. As a 30- to 40-mm square-shaped PGAs is gripped by grasping forceps through the endoscopic channel and delivered from the mouth to the stomach along with the endoscope, the PGAs is exposed to saliva and gastric juices, and it is difficult for the PGAs to maintain its sheet shape under such wet conditions. Once a PGAs becomes curled up in the stomach, it is very difficult if not impossible to uncurl the PGAs into its sheet form using an endoscope [9, 10]. This study aimed to evaluate the efficacy of appropriate and rapid PGAs coating method using device delivery station system (DDSS).

## Patients and methods

This study was conducted with approval from the ethics committees of the Ehime Rosai and Kagawa University Hospitals (Approval No. 80) and in accordance with the Declaration of Helsinki. The patients provided verbal and written informed consent. This trial was also registered with the University Hospital Medical Information Network (UMIN000026377) following the CONSORT check list.

**Fig. 1:**
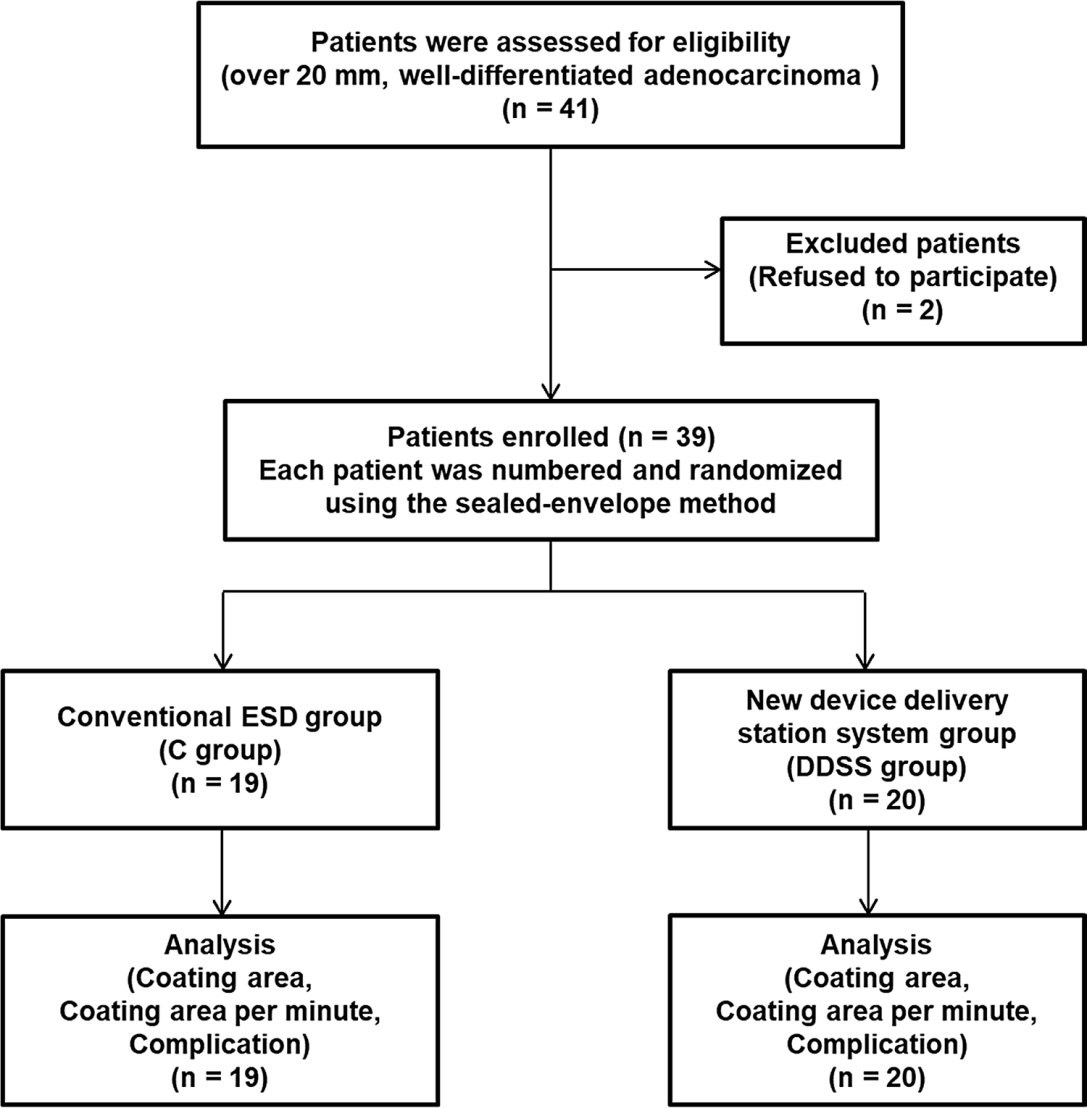
Flow chart of this study

Forty-one patients who were diagnosed with early gastric cancer over 20 mm in diameter by narrow band imaging magnified endoscopic examination (NBI-ME) and pathologically diagnosed with well-differentiated adenocarcinoma from biopsy specimens were eligible for this prospective randomized study. Two patients refused to participate and were excluded. The remaining 39 patients were randomly allocated using the sealed envelope method into the following two groups according to the delivery method: the conventional PGAs delivery group (*n* = 19) (C group) and the new DDSS group (*n* = 20) (DDSS group) (Fig. 1). Patients taking ticlopidine hydrochloride, clopidogrel sulfate or aspirin were switched to cilostazol 3 days before ESD, and they discontinued cilostazol the day of ESD. All antiplatelet drugs were resumed the day after ESD. The three endoscopists who performed ESD were members of the Japan Gastroenterological Endoscopy Society and received a lecture on the DDSS beforehand.

In the C group, a 50-mm square-shaped PGAs was gripped by grasping forceps through an endoscopic channel and delivered from the mouth to the stomach along with the endoscope. The PGAs coating time was defined as the duration from the beginning of endoscope insertion into the mouth to the end of the fibrin glue coating process.

In the DDSS group, a nasal endoscope with an attached DDSS was prepared beforehand following specified procedures. The PGAs coating time was defined the same as it was for the C group. In addition to several parameters measured during ESD, other calculations and analyses were conducted as follows:

The short axis (S) and long axis (L) (cm) of the ellipsoid dissected specimen were measured after ESD.

- The ellipsoid coating area was defined as the area calculated by the following formula: the ellipsoid coating area (cm^2^) = π × L/2 × S/2 (π = 3.14)
- The coating area per minute was calculated by the following formula: the coating area per minute (cm^2^/min) = the ellipsoid coating area (cm^2^)/the PGAs coating time (min)

### Outcomes

#### Primary outcome

1. Coating area per minute in the C group and DDSS group, respectively (cm^2^/min).

#### Secondary outcomes

2. Post-ESD bleeding (1–7 days after ESD): post-ESD bleeding was defined as bleeding that required an intervention with hemostatic forceps.
3. Post-ESD perforation (1–7 days after ESD).

### Sample size calculation

After we conducted a pilot study of five patients in the C group and five patients in the DDSS group (a total of ten patients), we found significant differences between the two groups in the coating area per minute (C) (C_C_ and C_DDSS_) (cm^2^/min). Based on these results, the sample size required for an α error of 0.05 and a power of 0.8 with a standard deviation of 0.0351 was calculated to be 20 patients by performing a statistical analysis using GraphPad Prism (http://biostat.mc.vanderbilt.edu/wiki/Main/PowerSampleSize).

### Devices

Endoscopes: GIF TYPE XP260NS (Olympus Co., Tokyo, Japan).

Incisional knife: Dual knife®(KD-650 L), IT knife 2®(KD-611L) (Olympus Co., Tokyo, Japan).

Incisional generator device: ERBE VIO300D (Elektromedizin, Tübingen, Germany).

CO_2_ insufflation device: OLYMPUS UCR (Olympus Co., Tokyo, Japan).

PGAs (Gunze Co., Kyoto, Japan).

### Procedures for using the PGAs DDSS (Videos 1 and 2)

#### Video 1

**Fig. 2:**
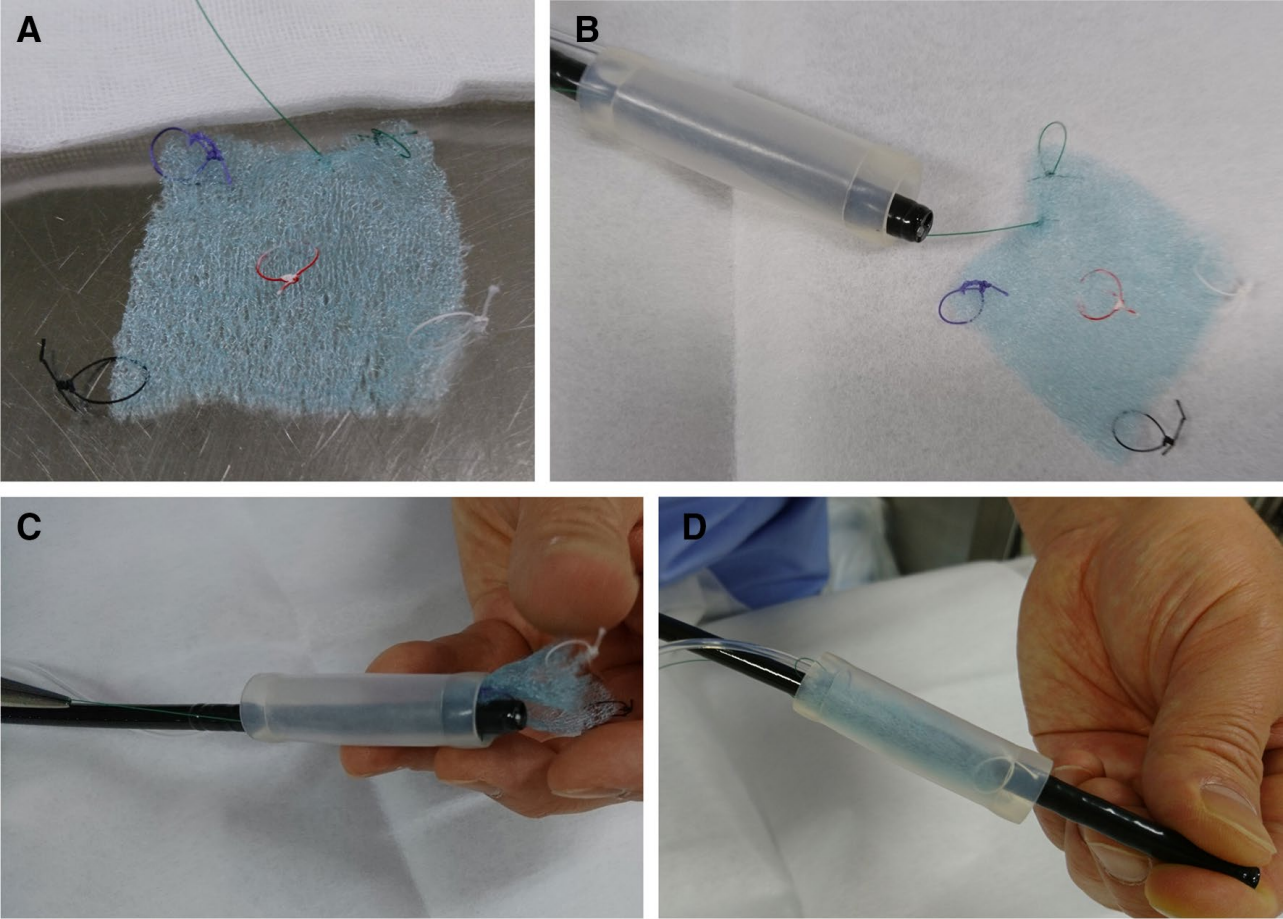
PGAs with ring-shaped threads of four different colors. **A** A PGAs was cut into a 50-mm square sheet. Ring-shaped threads 3–4 mm in diameter of four different colors were placed in the corners of a 50-mm square sheet. A ring-shaped thread was also placed in the center of PGAs. The location of each colored thread is important for recognizing the orientation of PGAs, especially if PGAs becomes wet and curled up in the stomach due to unpredicted factors. For example, the thread on the contralateral side to the black thread was green, and that to the white thread was purple. **B** A nasal endoscope was inserted into an endoscopic injection sclerotherapy (EIS) balloon, and the 5-cm thread on one side of the square PGAs was pulled through the EIS balloon. **C** PGAs was stored in the gap between the nasal endoscope and the EIS balloon by pulling the thread. **D** Then, PGAs was placed equally around the nasal endoscope

**Fig. 3:**
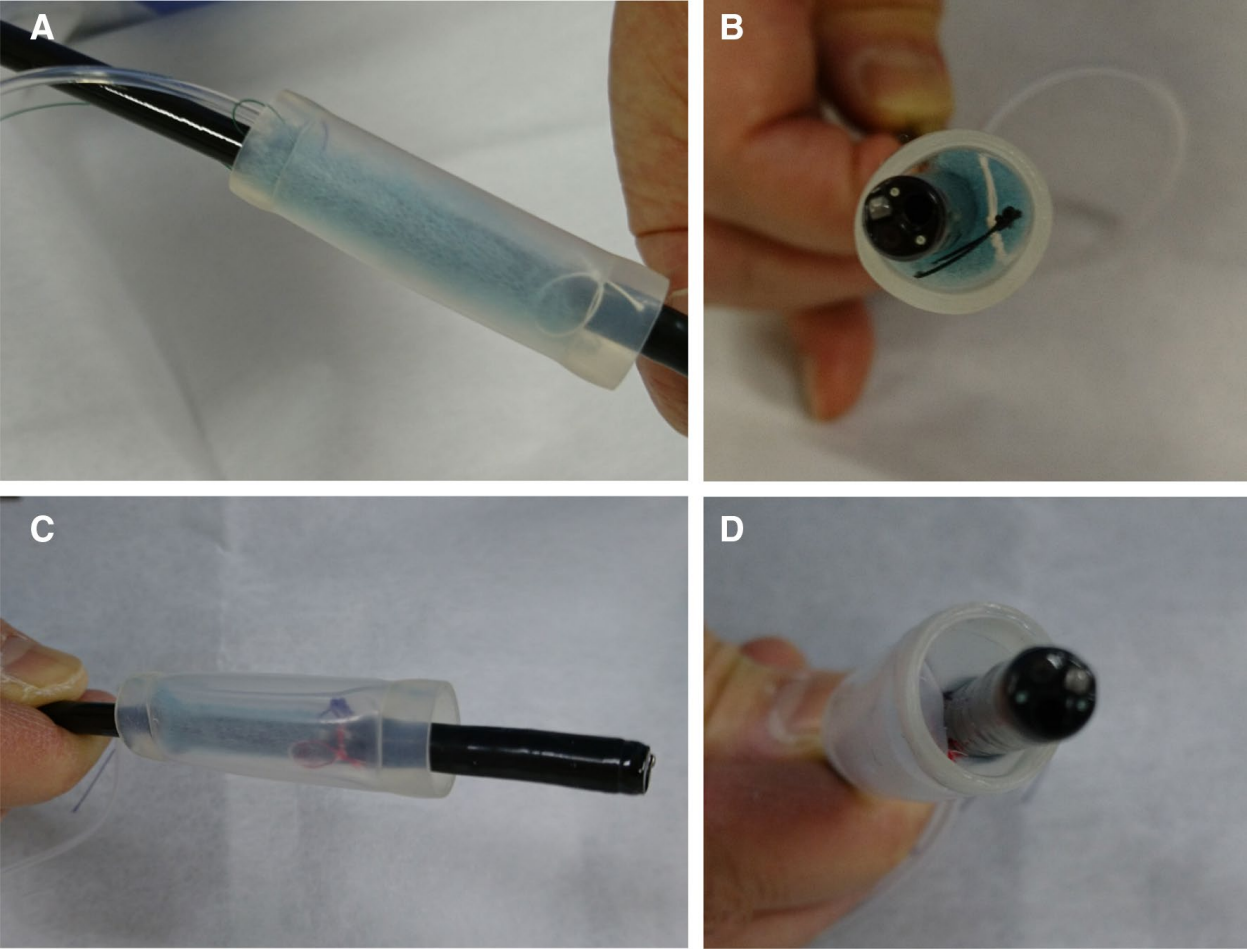
PGAs stored equally around the nasal endoscope by air insufflation. **A** Using tweezers, PGAs was adjusted to be placed equally around the nasal endoscope. **B** As viewed from in front of the endoscope tip, the PGAS was placed in the gap between the EIS and nasal endoscope. **C** As EIS balloons swell equally outward and inward, PGAs was sealed between the endoscope and the inner bulge of the swollen balloon, then, insufflating approximately 4.5 ml of air did not cause the outer balloon to swell and PGAs was sealed in the center of an EIS balloon. **D** Thus completing the preparation of the nasal endoscope with PGAs delivery system (NE-PGAs) was made

An endoscopic injection sclerotherapy (EIS) balloon (MD-47411L, 8 mm in diameter, 60 mm in length) (Sumitomo Bakelite Co., Tokyo, Japan) and a nasal endoscope (GIF TYPE XP260NS, Olympus Co., Tokyo, Japan) were prepared. Ring-shaped threads 3–4 mm in diameter of different colors were placed in each corner of the 50-mm PGAs. A 5-cm thread was attached to one side of the square PGAs and was used for housing PGAs in the EIS balloon (Fig. 2A). The nasal endoscope was inserted into the EIS balloon, and the 5-cm thread attached on PGAs was pulled through the EIS balloon (Fig. 2B). PGAs was stored in the gap between the nasal endoscope and the EIS balloon (Fig. 2C). PGAs was distributed equally around the nasal endoscope (Fig. 2D). PGAs was equally distributed around the nasal endoscope (Fig. 3A, B). Approximately 4.5 ml of air was insufflated, which did not cause outer EIS balloon swelling (Fig. 3C). Thus completing the preparation of the nasal endoscope with PGAs delivery system (NE-PGAs) was made (Fig. 3D).

#### Video 2

**Fig. 4:**
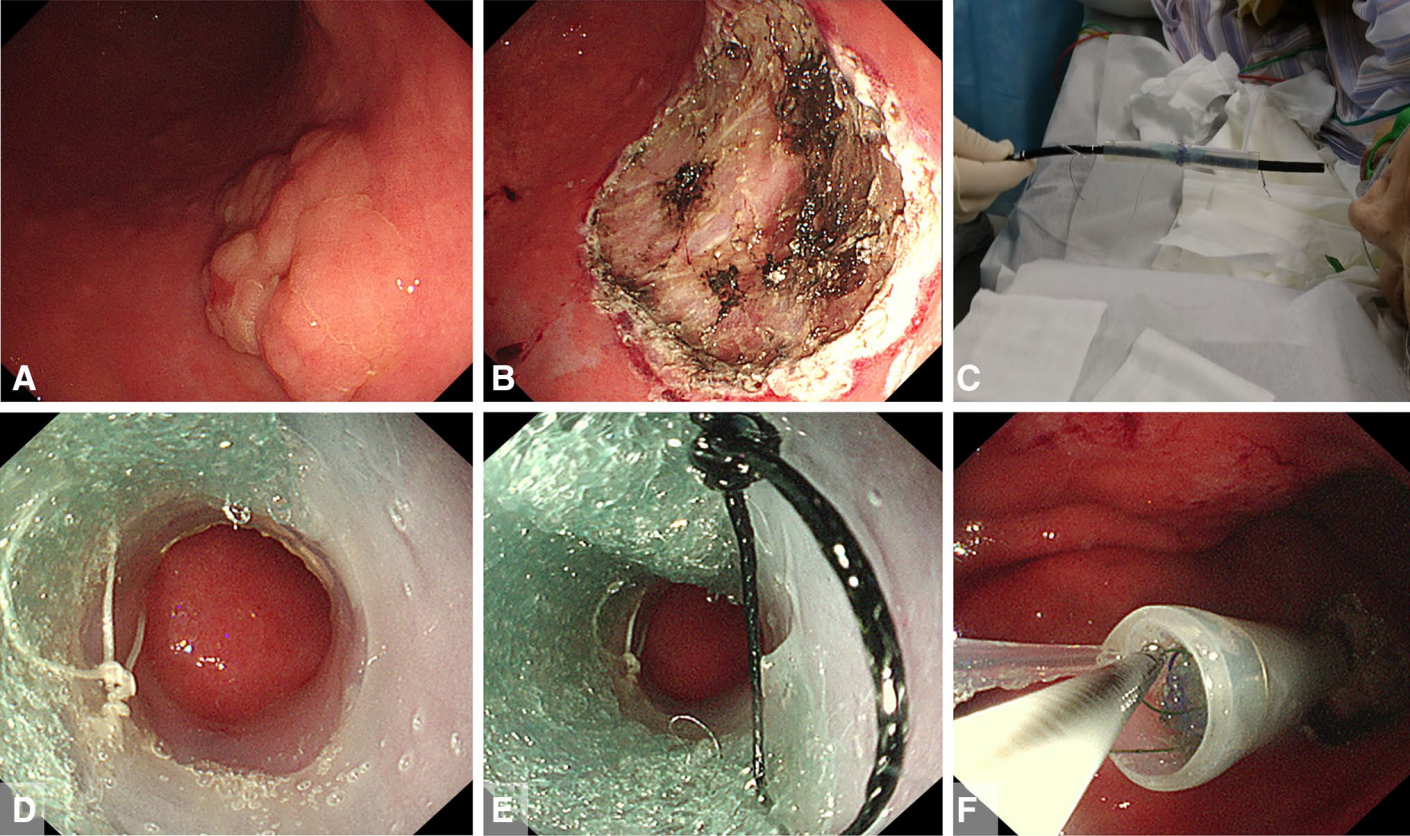
Procedures for delivering a PGAs using the DDSS. **A** Esophagogastroduodenoscopy revealed rather thick 0-IIa-type early gastric cancer 45 mm in diameter in the posterior wall of the body of the stomach. ESD was completed within 50 min with bleeding prevented by conducting pre-coagulation with coagulation forceps. **B** The post-ESD artificial ulcer became 50 mm in diameter. **C** A nasal endoscope with a PGAs (NE-PGAs) was inserted and passed from the mouth to the stomach. **D** After placing NE-PGAs in the stomach, 4.5 ml of air was deflated from the EIS balloon and the nasal endoscope was pulled out through the EIS balloon with PGAs. **E** By pulling out the nasal endoscope slowly, the white and black threads attached to PGAs corners could be observed. While the EIS balloon and PGAs remained in the stomach, the nasal endoscope was replaced by an oral endoscope to proceed with the treatment. **F** By grasping the thread attached to the corner of PGAs, PGAs was pulled out from NE-PGAs

**Fig. 5:**
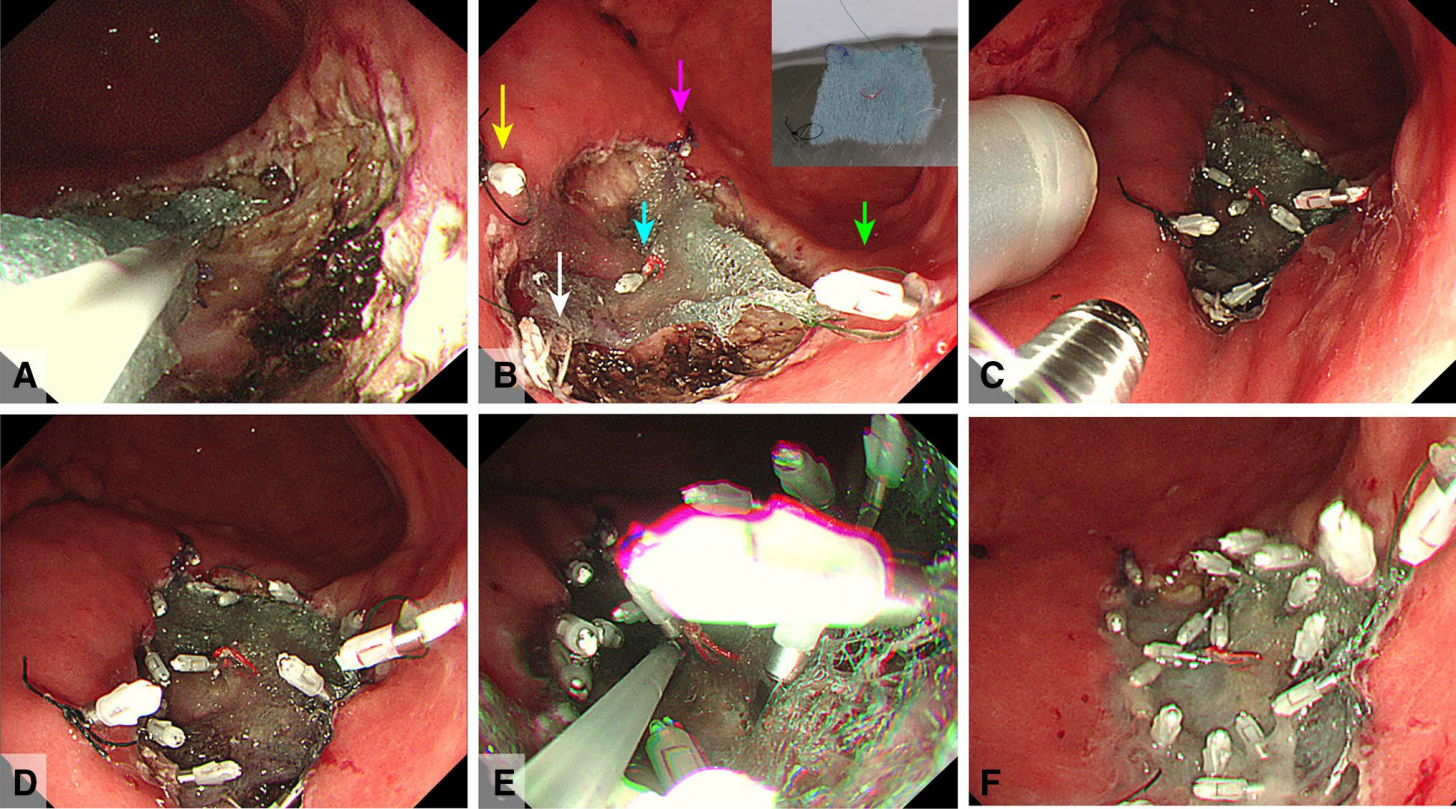
Tight PGAs attachment. **A** PGAs was brought to the post-artificial ulcer using forceps. **B** PGAs was unfolded by referencing the four threads attached to the corners and the one thread attached to the center of the PGAs (black thread: yellow arrow, white thread: white arrow, green thread: green arrow, purple thread: pink arrow, red thread: blue arrow). **C** PGAs was clipped and fixed equally on each of the four corners by referring to the four colored threads, and clipping the center of PGAs was very important for tightly attaching PGAs to the post-ESD artificial ulcer floor. **D** The periphery of PGAs was clipped and fixed equally just above the ulcer floor for tight attachment. Finally, the red thread at the center of PGAs was fixed at the center of the artificial ulcer. **E** Beriplast P Combi-Set Tissue Adhesive®(combination of fibrin glue and thrombin) was applied equally to the artificial ulcer. **F** PGAs was completely and tightly attached to the post-ESD artificial ulcer floor

Esophagogastroduodenoscopy revealed rather thick, 0-IIa-type, early gastric cancer 45 mm in diameter in the posterior wall of the body of the stomach (Fig. 4A). The post-ESD artificial ulcer became 50 mm in diameter (Fig. 4B). NE-PGAs was inserted from the mouth to the stomach (Fig. 4C). After 4.5 ml of air was deflated from the EIS balloon, the nasal endoscope was pulled out through the EIS balloon (Fig. 4D). By pulling out the nasal endoscope slowly, PGAs was observed (Fig. 4E). By grasping the thread, PGAs could be pulled out from the NE-PGAs (Fig. 4F). PGAs was brought to the post-ESD ulcer (Fig. 5A). PGAs was unfolded by referencing the four threads attached to the corners (Fig. 5B). All of the threads attached to PGAs were clipped and fixed equally (Fig. 5C). More clips were added just above the ulcer floor (Fig. 5D). Beriplast P Combi-Set Tissue Adhesive®(CSL Behring K.K., Tokyo, Japan) (combination of fibrin glue and thrombin) was applied equally to the artificial ulcer (Fig. 5E). The tight attachment of PGAs to the post-ESD artificial ulcer floor was completed (Fig. 5F).

### Statistical analysis

For comparing the relative frequencies between groups, data were analyzed using Fisher’s exact test or the *χ*^2^ test. The Mann–Whitney *U* test was used to compare continuous variables with a significance level of *p* < 0.05. Statistical analyses were performed using GraphPad Prism version 5.00 for Windows (GraphPad Software, San Diego, CA, USA).

## Results

There were no significant differences in patient age or gender (*p* = 0.66 and 0.848, respectively) between the C group and DDSS group. Regarding the location of lesions, in the C group (19 lesions), six were upper (U) lesions, seven were middle (M) lesions, and six were lower (L) lesions. In the DDSS group (20 lesions), five were U lesions, eight were M lesions, and seven were L lesions. There was no significant difference in lesion location (*p* = 0.209).

The macroscopic findings of lesions revealed that the 0-IIa, 0-IIc, and 0-IIa + IIc types accounted for 6, 4, and 9 lesions, respectively, in the C group. In the DDSS group, the 0-IIa, 0-IIc, and 0-IIa + IIc types accounted for 7, 5, and 8 lesions, respectively. There was no significant difference in the macroscopic findings (*p* = 0.479) (Table 1).

**Table 1:** Baseline characteristics

	C group (*n* = 19)	DDSS group (*n* = 20)	*p* value*
Age, years (median[range])	76 [52–91]	77 [49–93]	0.66*
Gender (male/female)	11/8	12/8	0.848**
Locations of lesions			0.209**
U	6	5	
M	7	8	
L	6	7	
Macroscopic findings of lesions			0.479***
0-IIa	6	7	
0-IIc	4	5	
0-IIa + IIc	9	8	
Oral administration of antithrombotic agents			
Anticoagulants	4	5	0.777*
Antiplatelets	3	4	0.740*
Aspirin/ticlopidine hydrochloride/clopidogrel sulfate	2/3/2	3/4/2	0.383****

*Unpaired *t* test, **χ^2^ test, ***Fisher’s exact test, ****non-repeated measures ANOVA test

*C group* conventional PGAs delivery group, * DDSS group* PGAs delivery group using device delivery station system

**Fig. 6:**
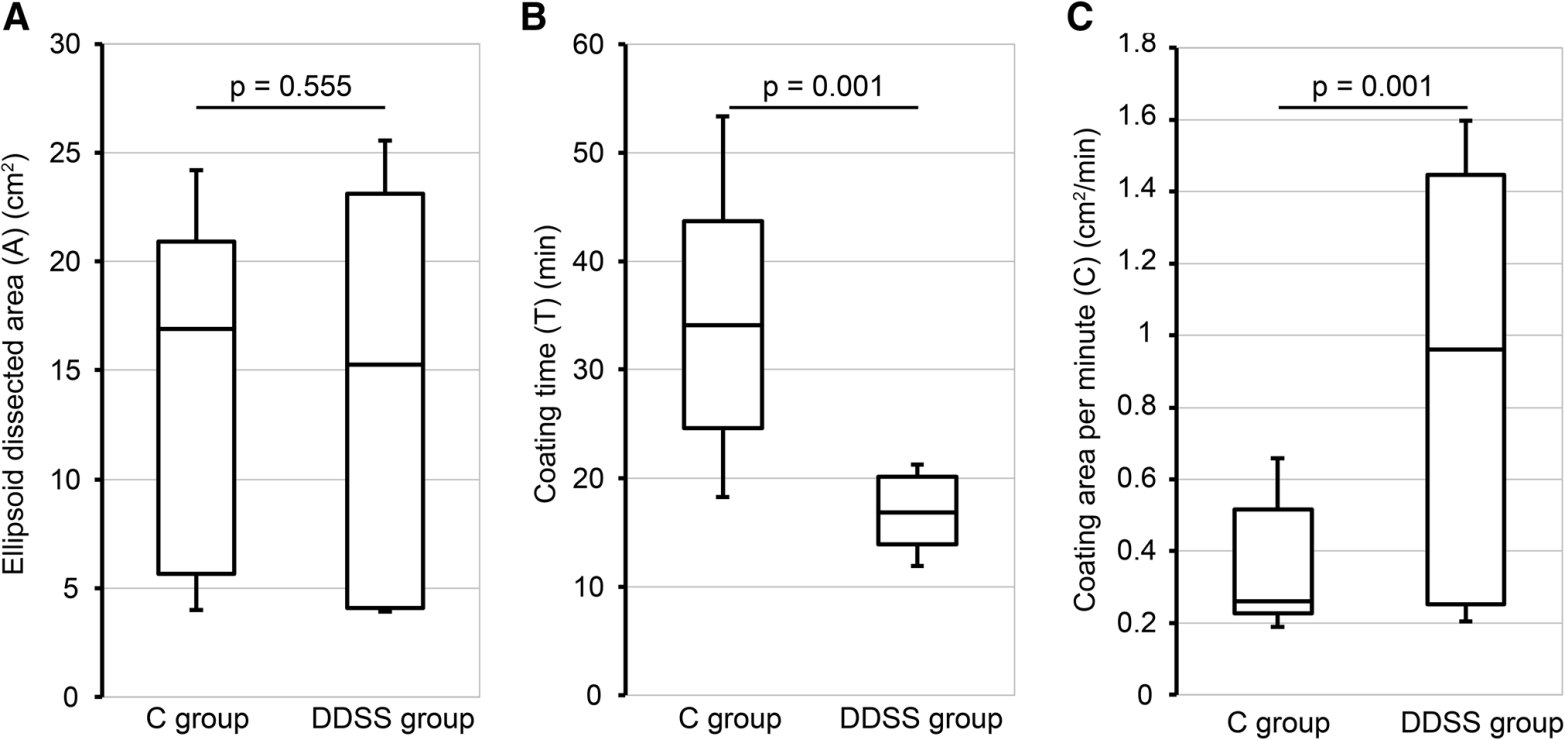
Results. **A** There were no significant differences in the ellipsoid dissected area (A) (cm^2^), with values of 16.9 (3.68–25.3) and 15.3 (3.56–27.2) cm^2^ for the C and DDSS groups, respectively (*p* = 0.555). **B** There were significant differences in the coating time (T) (min), with values of 34.1 (15.0–60.7) and 16.85 (11.5–27.2) min for the C and DDSS groups, respectively (*p* = 0.001). **C** There was a significant difference in the coating area per minute (C) (cm^2^/min), with values of 0.261 (0.02–1.00) and 0.96 (0.173–2.06) cm^2^/min for the C and DDSS groups, respectively (*p* = 0.001)

There was also no significant difference in the ellipsoid dissected area (cm^2^), with areas of 16.9 (3.68–25.3) cm^2^ and 15.3 (3.56–27.2) cm^2^ for the C group and DDSS group, respectively (*p* = 0.555) (Table 2) (Fig. 6A). There was a significant difference in the coating time (min), with values of 34.1 (15.0–60.7) and 16.85 (11.5–27.2) min for the C group and DDSS group, respectively (*p* = 0.001) (Fig. 6B). In the coating areas per minute (cm^2^/min), there was a significant difference, with values of 0.261 (0.02–1.00) cm^2^/min and 0.96 (0.173–2.06) cm^2^/min (*p* = 0.001) (Table 2) (Fig. 6C) for the C group and DDSS group, respectively.

**Table 2:** Results

	C group (*n* = 19)	DDSS group (*n* = 20)	*p* value*
Ellipsoid dissected area (cm^2^), median (range)	16.9 (3.68–25.3)	15.3 (3.56–27.2)	0.555*
Coating time (min), median (range)	34.1 (15.0–60.7)	16.85 (11.5–27.2)	0.001*
Coating area per minute (cm^2^/min), median (range)	0.261 (0.02–1.00)	0.96 (0.173–2.06)	0.001*
Post-ESD bleeding (1–7 days after ESD) (cases)	4 (21%)	0 (0%)	0.030*
Post-ESD perforation (1–7 days after ESD) (cases)	0	0	0.725*
PGAs remained POD 3, 7, 14, 30, and 60 in ulcer floor	4/2/1/0/0 (Total 7 patients)	1/2/5/0/0 (Total 8 patients)	0.0006**
Pathological invasion depth			0.593**
M	13	15	
Sm1	4	3	
Sm massive	2	2	

*Mann–Whitney *U* test, **Fisher’s exact test

*C group* conventional PGAs delivery group,* DDSS group* PGAs delivery group using device delivery station system

Anticoagulants were used in four and five patients in the C group and DDSS group, respectively (*p* = 0.777). Antiplatelet drugs were used in three and four patients in the C group and DDSS group, respectively (*p* = 0.740). There were no significant differences between the groups (Table 1).

**Fig. 7:**
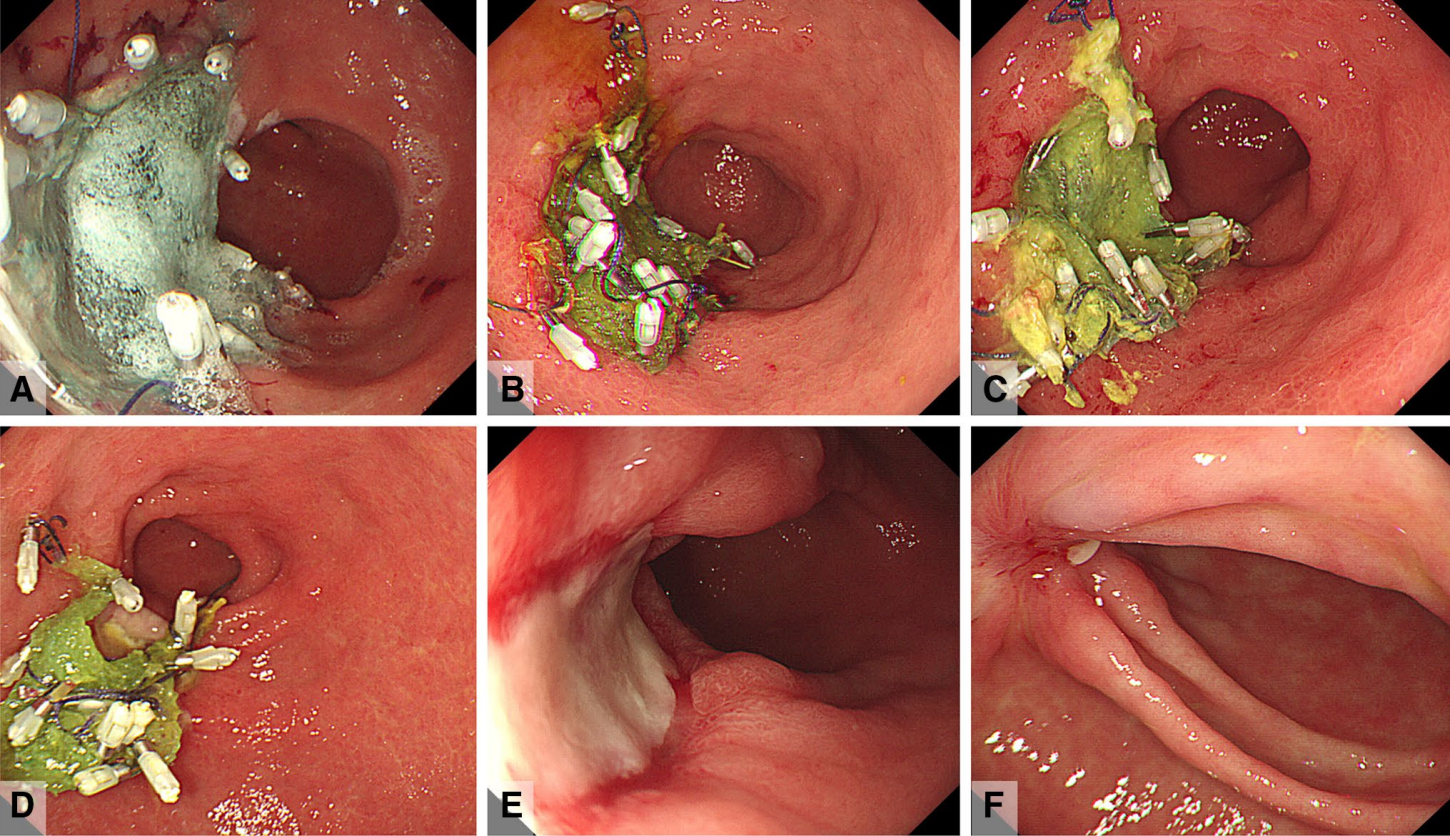
Typical healing course of the PGAs-coated post-ESD ulcer floor. **A** The PGAs-coated ulcer floor just after ESD. **B** The PGAs-coated ulcer floor on post-operation day (POD) 3. **C** The PGAs-coated ulcer floor on POD 7. **D** The PGAs-coated ulcer floor on POD 14. **E** The PGAs-coated ulcer floor on POD 30. **F** The PGAs-coated ulcer floor on POD 60

Among antiplatelet drugs, aspirin/ticlopidine hydrochloride/clopidogrel sulfate were taken by 2/3/2 patients in the C group and by 3/4/2 patients in the DDSS group, respectively. There was no significant difference in the use of antiplatelet drugs between the groups (*p* = 0.383) (Table 1). There were 4 (21% of C group) cases of post-ESD bleeding (1–7 days after ESD) in the C group compared with 0 in the DDSS group, which was significantly different (*p* = 0.030). There were 0 cases of perforation during ESD in both groups, with no significant difference (*p* = 0.725) (Table 2). Instances of post-ESD bleeding were successfully controlled with hemostatic forceps. Total seven patients in C groups and eight patients in DDSS groups allowed us to perform follow up EGD examination. Patients those PGAs remained until post-operational day (POD) 3, 7, 14, 30, and 60 were 4/2/1/0/0 patients in C groups, and 1/2/5/0/0 patients in DDSS groups with significant difference (*p* = 0.0006). The typical healing course of the PGAS-coated post-ESD ulcer floor in the DDSS group is shown in Fig. 7. Images were captured of the PGAS-coated ulcer floor just after ESD (Fig. 7A) and on POD 3 (Fig. 7B), POD 7 (Fig. 7C), POD 14 (Fig. 7D), POD 30 (Fig. 7E), and POD 60 (Fig. 7F).

Pathological examinations revealed no significant differences in early cancer invasion depth, i.e., mucosal, submucosal or sm massive, with 13, 4, and 2 cases in the C group and 15, 3, and 2 cases in the DDSS group showing mucosal and submucosal invasion depths, respectively (*p* = 0.593) (Table 2).

## Discussion

**Fig. 8:**
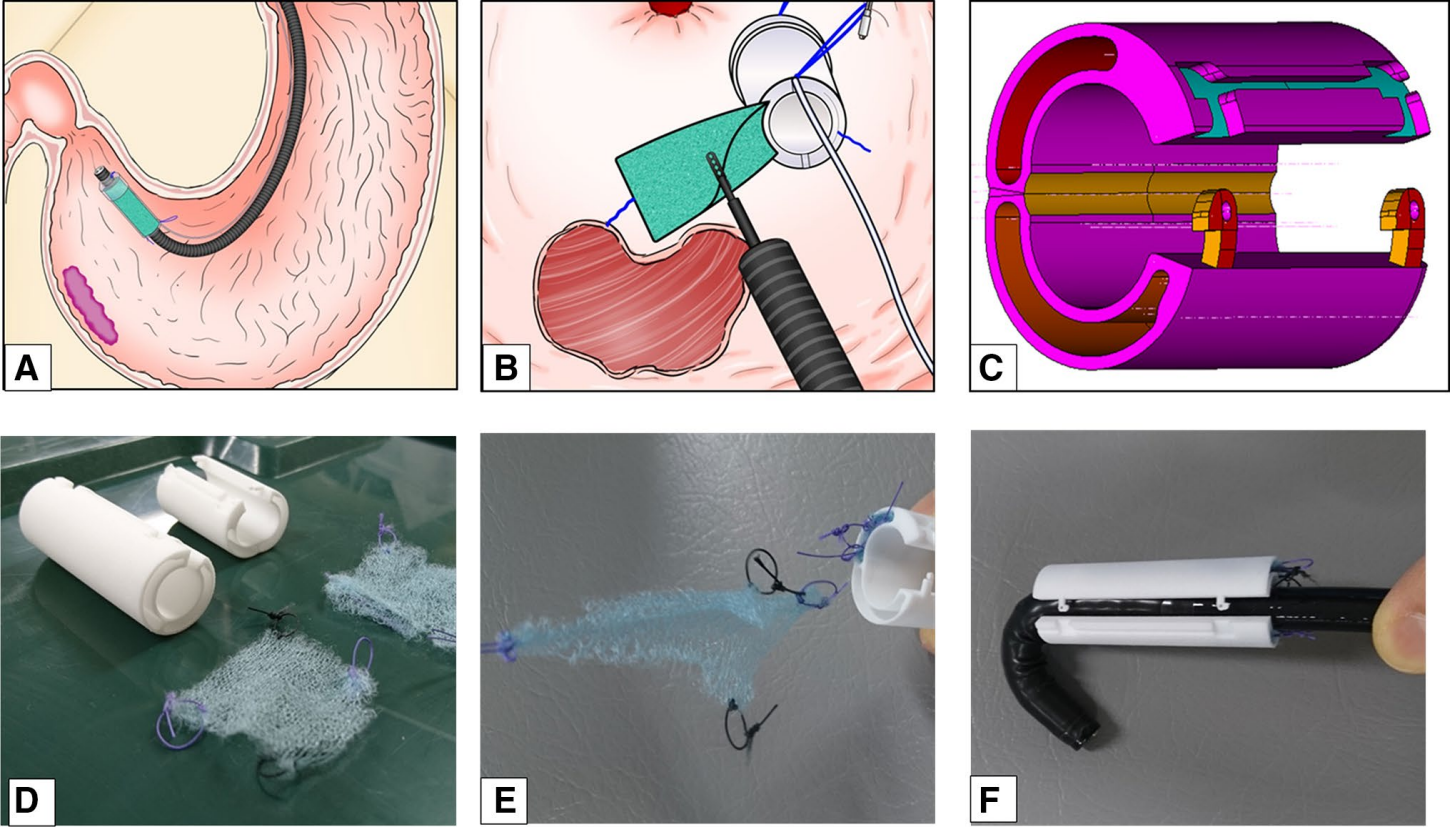
Concept and prototype of the endoscopic device delivery station system (E-DDSS). **A** The E-DDSS can deliver various endoscopic devices and place them within the digestive tract via a detachable functionality while maintaining completely sealed conditions. **B** E-DDSS can deliver various endoscopic devices and detained within the digestive tract by detachable function under completely sealed condition. **C** E-DDSS has two chambers in which PGAs were stored, and it was delivered by splitting one side of the device by unlocked system. **D** Two E-DDSS prototypes (3 or 5 cm in length). **E** From independent E-DDSS attached to the endoscope, it is possible to pull out loaded items, such as hemostatic gauze or a PGAs. **F** The E-DDSS can be attached to an oral endoscope or a nasal endoscope

PGAs are made of PGA, which is widely used in bio-absorbable sutures in the surgical field. During the healing process of a post-ESD artificial ulcer, a PGAs exerts anti-inflammatory effects and prompts the creation of rich granulation tissue. Simultaneously, the PGAs prompts the migration of epidermal cells over the rich granulation tissue and accelerates the activity of fibroblasts to form collagenous tissue with a scar. After these processes, the PGAs becomes almost completely absorbed within approximately 3 months (15 weeks). Early anti-inflammatory effects and rich granulation tissue formation not only prevent deformation of the post-ESD artificial ulcer by fibroblasts but also play a role in protecting the ulcer floor from exogenous materials and factors [11]. These protective mechanisms seem to be very similar to reactions induced by steroid local injection therapy to prevent stenosis or deformation of the esophagus or stomach [12–14]. Although a PGAs has no inherent hemostatic activity, covering the ulcer floor with a PGAs and coating the covering with fibrin glue provide protection, deformity prevention, and hemostatic effects. The tight attachment of a PGAs to the artificial floor is necessary to obtain a sufficient hemostatic effect; insufficient attachment leads to post-ESD bleeding. PGAs is very useful for covering post-ESD ulcer floors to provide protection from exogenous materials and hemostasis with an additional fibrin glue coating [15, 16]. On the other hand, there are two major problems in applying a PGAs to a post-ESD ulcer floor, as follows: ① rapidly delivering the thin PGAs without exposing it to saliva or gastric juice [10]; and ② tightly attaching the PGAs to the ulcer floor using only a flexible endoscope such that a sufficient hemostatic effect is obtained [9]. For ①, we have developed a novel endoscopic device delivery concept and produced a prototype of the innovative device, i.e., the E-DDSS (Fig. 8A). The E-DDSS can deliver various endoscopic devices that can be placed within the digestive tract via a detachable functionality under completely sealed conditions (Fig. 8B). E-DDSS has two chambers in which PGAs were stored, and it was delivered by splitting one side of the device by unlocked system (Fig. 8C). There were two types of E-DDSS prototypes (3 or 5 cm in length) (Fig. 8D). From the E-DDSS attached to the endoscope, it is possible to pull out items loaded in the circumferential cavities, such as hemostatic gauze or a PGAs (Fig. 8E). The E-DDSS can be attached to an oral endoscope or a nasal endoscope (Fig. 8F). The E-DDSS is now being tested in several in vivo experiments. As a DDSS that can deliver a PGAs is included within one of the concepts of the E-DDSS, in this study, a DDSS that used an existing EIS balloon was used to deliver a PGAs, and this approach yielded better results than the conventional delivery method. However, using the DDSS with a nasal endoscope for PGAs delivery (NE-PGAs) required the immediate replacement of the nasal endoscope by an oral endoscope, which slightly increased the procedure duration [10]. For ②, tight PGAs attachment was achieved by using a PGAs with four ring-shaped colored threads placed in the four corners of the sheet to more easily recognize the orientation of the PGAs and facilitate sheet fixation with hemoclips. Post-ESD bleeding is one of the crucial events especially for patients who take antiplatelet agents. There were some reports that post-bleeding events occurred within 7–14 days after ESD. Follow-up EGDs until POD 60 revealed PGAs remained on POD 7–14 in almost of all patients without post-ESD bleeding events. Using a PGAs with four ring-shaped colored threads also enabled the effective, tight attachment of the PGAS such that post-ESD bleeding was decreased among patients using antithrombotic agents [17–19]. In our study, with limitation of small number, although PGAs prevented post-ESD bleeding, it could not prevent the deformity of post-ESD ulcer. Although there were several reports that PGAs prevented stricture of esophagus after ESD [3, 11], we could not confirm anti-deformity effect of PGAs. Considering the healing process, PGAs prompts the migration of epidermal cells and accelerates the activity of fibroblasts to form collagenous tissue with a scar, and these effects might be able to prevent post-ESD bleeding, but not deformity of stomach. Further multi-center studies are needed to confirm the main effect of PGAs under the same coating method like DDSS.

In conclusion, this DDSS was very useful for rapidly delivering and tightly attaching a PGAs to control post-ESD bleeding.

## Electronic supplementary material

Below is the link to the electronic supplementary material.

How to make PGAs device delivery station system. An endoscopic injection sclerotherapy (EIS) balloon and a nasal endoscope were prepared. PGAs was cut into a 50-mm square sheet. One ring-shaped thread 3–4 mm in diameter was placed in each corner of the sheet, and each thread was a different color. The location of each colored thread is important for recognizing the orientation of PGAs. For example, the thread on the contralateral side to the black thread was green, and that to the white thread was purple. A 5-cm thread was attached to one side of the square PGAs and was used for housing the PGAs in the EIS balloon. PGAs was stored in the gap between the nasal endoscope and the EIS balloon. Approximately 4.5 ml of air was insufflated into EIS balloon. PGAs was sealed in the center of an EIS balloon. (ZIP 39262 KB)

Tight attachment of PGAs. Esophagogastroduodenoscopy revealed 0-IIa-type, early gastric cancer 45 mm in diameter in the posterior wall of the stomach. The post-ESD ulcer became 50 mm in diameter. NE-PGAs was inserted from the mouth to the stomach. After 4.5 ml of air was deflated from the EIS balloon, the nasal endoscope was pulled out. By grasping the thread attached to the corner of PGAs, PGAs was brought to the post-ESD ulcer using forceps. After the white thread was fixed at the proximal side of the ulcer edge, the black thread was fixed at the greater curvature. As the contralateral corner of the black thread was green, the green thread was fixed at the lesser curvature. In the same manner, the purple thread at the contralateral corner of the white thread was fixed at the distal side. Finally, the red thread at the corner of the PGAs was fixed at the center of the ulcer. More clips were added just above the ulcer floor to fix the periphery of the PGAs equally for tight attachment. Beriplast P®(combination of fibrin glue and thrombin) was applied equally to the artificial ulcer. The tight attachment of the PGAs to the post-ESD ulcer floor was completed. (ZIP 59805 KB)

## References

[1] Maekawa S, Nomura R, Murase T, Ann Y, Harada M (2015). Complete closure of artificial gastric ulcer after endoscopic submucosal dissection by combined use of a single over-the-scope clip and through-the-scope clips (with videos). Surg Endosc.

[2] Takimoto K, Toyonaga T, Matsuyama K (2012). Endoscopic tissue shielding to prevent delayed perforation associated with endoscopic submucosal dissection for duodenal neoplasms. Endoscopy.

[3] Sakaguchi Y, Tsuji Y, Ono S, Saito I, Kataoka Y, Takahashi Y, Nakayama C, Shichijo S, Matsuda R, Minatsuki C, Asada-Hirayama I, Niimi K, Kodashima S, Yamamichi N, Fujishiro M, Koike K (2015). Polyglycolic acid sheets with fibrin glue can prevent esophageal stricture after endoscopic submucosal dissection. Endoscopy.

[4] Doyama H, Tominaga K, Yoshida N, Takemura K, Yamada S (2014). Endoscopic tissue shielding with polyglycolic acid sheets, fibrin glue and clips to prevent delayed perforation after duodenal endoscopic resection. Dig Endosc.

[5] Takimoto K, Imai Y, Matsuyama K (2014). Endoscopic tissue shielding method with polyglycolic acid sheets and fibrin glue to prevent delayed perforation after duodenal endoscopic submucosal dissection. Dig Endosc.

[6] Tsuji Y, Ohata K, Gunji T, Shozushima M, Hamanaka J, Ohno A, Ito T, Yamamichi N, Fujishiro M, Matsuhashi N, Koike K (2014). Endoscopic tissue shielding method with polyglycolic acid sheets and fibrin glue to cover wounds after colorectal endoscopic submucosal dissection (with video). Gastrointest Endosc.

[7] Takao T, Takegawa Y, Shinya N, Tsudomi K, Oka S, Ono H (2015). Tissue shielding with polyglycolic acid sheets and fibrin glue on ulcers induced by endoscopic submucosal dissection in a porcine model. Endosc Int Open.

[8] Ono H, Takizawa K, Kakushima N, Tanaka M, Kawata N (2015). Application of polyglycolic acid sheets for delayed perforation after endoscopic submucosal dissection of early gastric cancer. Endoscopy.

[9] Takao T, Takegawa Y, Ono H, Takao M, Oka S, Shinya N, Kutsumi H, Azuma T (2017). A novel and effective delivery method for polyglycolic acid sheets to post-endoscopic submucosal dissection ulcers. Endoscopy.

[10] Mori H, Kobara H, Rahman A, Nishiyama N, Nishiyama A, Suzuki Y, Masaki T (2017). Innovative delivery method using a detachable device to deliver a large polyglycolic acid sheet to a gastric ulcer perforation.. Endoscopy.

[11] Iizuka T, Kikuchi D, Yamada A, Hoteya S, Kajiyama Y, Kaise M (2015). Polyglycolic acid sheet application to prevent esophageal stricture after endoscopic submucosal dissection for esophageal squamous cell carcinoma. Endoscopy.

[12] Hashimoto S, Kobayashi M, Takeuchi M, Sato Y, Narisawa R, Aoyagi Y (2011). The efficacy of endoscopic triamcinolone injection for the prevention of esophageal stricture after endoscopic submucosal dissection. Gastrointest Endosc.

[13] Hanaoka N, Ishihara R, Takeuchi Y, Uedo N, Higashino K, Ohta T, Kanzaki H, Hanafusa M, Nagai K, Matsui F, Iishi H, Tatsuta M, Ito Y (2012). Intralesional steroid injection to prevent stricture after endoscopic submucosal dissection for esophageal cancer: a controlled prospective study. Endoscopy.

[14] Mori H, Rafiq K, Kobara H, Fujihara S, Nishiyama N, Kobayashi M, Himoto T, Haba R, Hagiike M, Izuishi K, Okano K, Suzuki Y, Masaki T (2012). Local steroid injection into the artificial ulcer created by endoscopic submucosal dissection for gastric cancer: prevention of gastric deformity. Endoscopy.

[15] Tsuji Y, Fujishiro M, Kodashima S, Ono S, Niimi K, Mochizuki S, Asada-Hirayama I, Matsuda R, Minatsuki C, Nakayama C, Takahashi Y, Sakaguchi Y, Yamamichi N, Koike K (2015). Polyglycolic acid sheets and fibrin glue decrease the risk of bleeding after endoscopic submucosal dissection of gastric neoplasms (with video). Gastrointest Endosc.

[16] Ono S, Tsuji Y, Fujishiro M, Kodashima S, Yamamichi N, Koike K (2014). An effective technique for delivery of polyglycolic acid sheet after endoscopic submucosal dissection of the esophagus: the clip and pull method. Endoscopy.

[17] Shindo Y, Matsumoto S, Miyatani H, Yoshida Y, Mashima H (2016). Risk factors for postoperative bleeding after gastric endoscopic submucosal dissection in patients under antithrombotics. World J Gastrointest Endosc.

[18] Matsumura T, Arai M, Maruoka D, Okimoto K, Minemura S, Ishigami H, Saito K, Nakagawa T, Katsuno T, Yokosuka O (2014). Risk factors for early and delayed post-operative bleeding after endoscopic submucosal dissection of gastric neoplasms, including patients with continued use of antithrombotic agents. BMC Gastroenterol.

[19] Fukuda H, Yamaguchi N, Isomoto H, Matsushima K, Minami H, Akazawa Y, Ohnita K, Takeshima F, Shikuwa S, Nakao K (2016). Polyglycolic acid felt sealing method for prevention of bleeding related to endoscopic submucosal dissection in patients taking antithrombotic agents. Gastroenterol Res Pract.

